# The genetic consequences of captive breeding, environmental change and human exploitation in the endangered peninsular pronghorn

**DOI:** 10.1038/s41598-022-14468-4

**Published:** 2022-07-04

**Authors:** Anastasia Klimova, Jesus Neftalí Gutiérrez-Rivera, Victor Sánchez-Sotomayor, Joseph Ivan Hoffman

**Affiliations:** 1 ACTG Molecular Solutions, 23085 La Paz, BCS Mexico; 2 Centro de Investigaciones Biológicas del Noroeste S.C., 23205 La Paz, BCS Mexico; 3 National Commission of Natural Protected Areas (CONANP), Valle de los Cirios Flora and Fauna Protection Area, Mexico; 4 Department of Animal Behaviour, Bielefeld University, Postfach 100131, 33501 Bielefeld, Germany; 5 British Antarctic Survey, High Cross, Madingley Road, Cambridge, CB3 OET UK

**Keywords:** Inbreeding, Genetic variation, Conservation biology

## Abstract

Endangered species with small population sizes are susceptible to genetic erosion, which can be detrimental to long-term persistence. Consequently, monitoring and mitigating the loss of genetic diversity are essential for conservation. The Peninsular pronghorn (*Antilocapra americana peninsularis*) is an endangered pronghorn subspecies that is almost entirely held in captivity. Captive breeding has increased the number of pronghorns from 25 founders in 1997 to around 700 individuals today, but it is unclear how the genetic diversity of the captive herd may have changed over time. We therefore generated and analysed data for 16 microsatellites spanning 2009–2021. We detected a decline in heterozygosity and an increase in the proportion of inbred individuals over time. However, these trends appear to have been partially mitigated by a genetically informed breeding management attempt that was implemented in 2018. We also reconstructed the recent demographic history of the Peninsular pronghorn, revealing two sequential population declines putatively linked to the desertification of the Baja California peninsula around 6000 years ago, and hunting and habitat loss around 500 years ago, respectively. Our results provide insights into the genetic diversity of an endangered antelope and indicate the potential for genetically informed management to have positive conservation outcomes.

## Introduction

Many species have experienced severe declines over the past two centuries as a result of growing anthropogenic pressures including direct exploitation, habitat destruction and climate change^1–3^. Some authors have even argued that Earth’s biodiversity is entering a sixth mass extinction event, characterised by the unprecedented loss of diversity at all levels^4–6^. Consequently, nowadays the persistence of many species is critically dependent on intensive management actions such as captive breeding, habitat restoration and reintroduction programs.

For many species, captive management has been the only option for persistence^7^. For example, species like the Kakapo (*Strigops habroptilus*), Przewalski’s horse (*Equus przewalskii*) and giant Galapagos tortoise (*Chelonoidis niger*), among many others, would have gone extinct without human intervention and ex situ management^8–10^. Captive breeding is frequently used for the preservation of threatened species and, in some cases, for the rehabilitation of declining populations^11–14^. However, it can sometimes inadvertently lead to genetic or behavioural changes that are not always beneficial^15^. For example, when selective pressures in captivity differ to those that are usually encountered by a species in the wild, maladaptive alleles or behaviours can rise to high frequency in captive populations, which can compromise the survival of individuals after they are reintroduced into the wild^16,17^. Furthermore, in small captive populations, strong genetic drift and the increased probability of mating between close relatives can decrease genome-wide heterozygosity and lead to inbreeding depression^18–22^. The fitness costs associated with inbreeding have been documented across taxonomic groups and include negative effects on litter size, longevity, female reproduction, male fertility and weight, in addition to hereditary defects^23–26^, all of which can have a strong impact on population viability.

Given that conserving genetic diversity and minimising inbreeding are important goals of most if not all captive breeding programmes^27,28^ and reduced genetic diversity has been associated with increased extinction risk and reduced adaptive potential^29–31^, knowledge of the effects of captive breeding on genetic diversity is crucial. In this regard, time-series genetic data from captive populations can be particularly useful^32–35^, as they can shed light on changes in key genetic characteristics of a population such as allelic richness, heterozygosity and the effective population size (*N*_e_); measures that reflect a combination of the speed of allele frequency change through genetic drift, the efficacy of selection and expected genetic diversity levels for selectively neutral loci^36,37^.

The pronghorn (*Antilocapra americana*) is the only extant species of the North American family Antilocapridae^38,39^. Pronghorns are thought to have been historically abundant, with documents from the 1800s suggesting that roughly 30–40 million individuals inhabited North America prior to the westward settlement of humans on the continent^40,41^. Nevertheless, current pronghorn numbers have been severely affected by habitat fragmentation and overhunting, with many populations having declined or disappeared entirely^42–44^. Nowadays, four pronghorn subspecies are recognized: the American pronghorn (*A. a. americana*), the Sonoran pronghorn (*A. a. sonoriensis*), the Peninsular pronghorn (*A. a. peninsularis*) and the Mexican pronghorn (*A. a. mexicana*)^45^. The American pronghorn is the most widespread subspecies, with the Sonoran, Peninsular and Mexican subspecies occupying more peripheral southern areas^40,44,45^. Of these subspecies, the Peninsular and Sonoran are currently under national and international protection^46–48^. Overall, the pronghorn is one of the many species currently undergoing captive breeding and translocation, with independent breeding programs active in the USA and Mexico^49–51^.

As with all of the pronghorn subspecies, wild populations of the Peninsular pronghorn have declined substantially since the arrival of the fist Spanish settlers^51^. By the beginning of the twentieth century, the Peninsular pronghorn was thought to number fewer than 1000 individuals^40^. These numbers have since fallen to fewer than a hundred individuals in the 1980s^42,51–53^. In the face of imminent extinction, a captive breeding program was established by the Peninsular Pronghorn Species Recovery Programme^51^. This commenced in 1997 at the Vizcaino Biosphere Reserve, Mexico, with 25 wild-caught adults and fawns being introduced to the breeding facilities during the first six years of the programme^49^. Since then, the captive herd has experienced steady growth, with around 60–100 young individuals being incorporated every year. Although systematic censuses have not been performed, the herd is known to have grown to around 198 individuals in 2006 and 250 individuals in 2010. Furthermore, some additional individuals were translocated to the USA and a number of animals also escaped captivity, with an aerial survey documenting a wild herd of 133 individuals in 2020. Therefore, the Peninsula pronghorn conservation programme represents a good example of a successful ongoing species recovery initiative in Mexico.

Currently, the animals are held in three management stations, with an additional six small populations held by a consortium of zoos in the Southwestern USA^53^. The main conservation area encompasses over 54,000 ha located in two protected natural areas: the El Vizcaíno Biosphere Reserve and the Valle de los Cirios Flora and Fauna Protection Area. Some of the individuals are allowed to roam freely over the protected areas and are provided only with supplementary feeding and water during the dry season^53^. Other animals, mainly the breeding herd and pregnant females, are managed in four smaller pens with year-round supplemental food and water, which are protected from predators by anti-coyote fencing.

In 2018, a genetically informed breeding management attempt was undertaken. A random selection of young but sexually mature males and females was microsatellite genotyped (2018 cohort, this paper) and a breeding plan was developed that focused on minimizing the relatedness of potential partners. Group-based management was implemented at one of the pens. At this pen, which consisted of only breeding females, two sexually mature males, selected on the basis of molecular estimates of kinship (and the possession of rare alleles), were introduced and kept there until the following year^35^. As not all of the individuals in the pen were sampled, some females with unknown relationships to the introduced males were also allowed to breed. Currently, the management team is looking to expand this strategy to include other management units and additional pens.

Previous population genetic studies of pronghorns reported moderate to high levels of genetic diversity in the American subspecies^45,54–57^, while genetic diversity appears to be somewhat lower for the Sonoran^54,58^ and Peninsular pronghorn subspecies^58,59^. Moreover, the American subspecies shows little evidence of population genetic structure^57^ while population genetic differentiation at the subspecies level is more pronounced, revealing clear genetic discontinuities between geographically isolated populations^35,58,59^.

The reasons for the relatively low genetic diversity of the Peninsular pronghorn subspecies are unknown, with two (non-mutually exclusive) explanations being possible. The first of these is that human induced habitat loss, competition with domestic animals and uncontrolled hunting may have caused the Peninsular pronghorn to decline over the past three centuries^44,51^, which may have been further exacerbated by small population sizes and inbreeding over the past few decades of captivity. Alternatively, or additionally, dramatic ecological changes during the last glacial maximum (LGM; ca. 12,000 years ago) resulted in the desertification of most of the Baja California peninsula, reducing water availability^60–62^ and likely contributing to a gradual reduction in pronghorn numbers over thousands of years.

Here, we generated a time series dataset of multilocus microsatellite data for the captive Peninsular pronghorn spanning the period 2009–2021 inclusive. We first evaluated changes in genetic diversity, heterozygosity and inbreeding over the past 13 years. We then used approximate Bayesian computation^63^ to evaluate support for alternative demographic scenarios that could explain the low genetic diversity of the Peninsular pronghorn, and to estimate relevant parameters such as the current *N*_e_ and the strength and timing of historical declines. We hypothesised that the collapse of the Peninsular pronghorn may have been driven by a combination of historical ecological changes and more recent anthropogenic pressures. We furthermore hypothesised that, although the captive breeding programme has been successful in increasing the number of individuals, there may have been some unavoidable loss of genetic diversity and an increase in inbreeding over time, although we expected that some of these changes might have been mitigated by the breeding management attempt.

## Results

### Summary statistics

We genotyped 144 pronghorn individuals at 16 microsatellite loci. Our genotyping error rate, estimated from 12 samples genotyped at eight loci, was 0.03 per locus. The overall rate of missing data was 6.1%, which fell to 4.6% when seven individuals with missing data at four loci were excluded. All of the multilocus genotypes were unique, indicating that no individuals had been inadvertently sampled more than once (e.g. initially as fawns and later as adults). Analysing the full dataset of 124 individuals (only fawns were included for 2012, 2016 and 2021; only adults were included for 2009 and 2018, Supplementary Table S1 online), we found significant deviations from Hardy–Weinberg equilibrium (HWE) at six loci (Supplementary Table S2 online). However, when the dataset was partitioned by year, we did not detect any consistent patterns of deviation from HWE across loci (Supplementary Table S2 online). Similar results were obtained for null alleles, with two loci (Aam1 and Anam6) showing indications of the presence of null alleles when all of the data were analysed together, while no consistent patterns were obtained when the years were analysed separately (Supplementary Table S3 online). Significant linkage disequilibrium (LD) was also detected for the full dataset (*p* = 0.02) but when the cohorts were analysed separately this was only present in 2009 (*p* = 0.01, Supplementary Table S4 online). Consequently, we retained all of the microsatellite loci for subsequent analyses.

### Genetic diversity

Among 124 captive Peninsular pronghorn individuals genotyped at 16 microsatellite loci, we detected a total of 88 alleles, with the mean number of alleles per locus being 5.5 (Table 1; Supplementary Table S5 online). Observed heterozygosity (*H*_*o*_) was slightly but not significantly (Bartlett’s K-squared = 0.071, *p* = 0.79) lower than expected heterozygosity (*H*_*e*_, Table 1; Supplementary Table S5 online). No significant differences among years were found for the basic diversity estimates (Supplementary Fig. S1 online), although *H*_*o*_ and *H*_e_ showed a weak tendency to decline over time, with the highest values being observed in 2009 and the lowest values being observed in 2018 (Supplementary Fig. S1).

**Table 1:** Mean values and standard errors (in parentheses) of genetic diversity estimates for the captive peninsular pronghorn based on 16 microsatellite loci.

Diversity index	Year					Full dataset
	2009	2012	2016	2018	2021	
*N*	18	33	45	19	9	124
*A*	3.4 (0.32)	3.8 (0.37)	3.8 (0.48)	3.7 (0.43)	3.0 (0.30)	5.5 (0.7)
*A*_r_	2.85 (0.22)	2.99 (0.22)	2.59 (0.17)	2.75 (0.22)	2.66 (0.24)	2.8 (0.09)
*H*_e_	0.51 (0.04)	0.53 (0.03)	0.46 (0.03)	0.45 (0.04)	0.46 (0.05)	0.51 (0.03)
*H*_o_	0.55 (0.05)	0.48 (0.04)	0.46 (0.03)	0.42 (0.04)	0.46 (0.07)	0.47 (0.02)
IR	0.10 (0.05)	0.25 (0.03)	0.18 (0.03)	0.32 (0.05)	0.38 (0.06)	0.22 (0.02)
sMLH	1.13 (0.07)	0.98 (0.05)	0.95 (0.04)	0.85 (0.04)	0.82 (0.08)	0.96 (0.02)
HL	0.44 (0.03)	0.52 (0.02)	0.55 (0.01)	0.59 (0.02)	0.61 (0.04)	0.54 (0.01)
TrioML	0.07 (0.02)	0.13 (0.02)	0.09 (0.01)	0.18 (0.03)	0.13 (0.02)	0.12 (0.01)

Genetic diversity was evaluated for the complete dataset of 124 individuals as well as separately for each year. *N* = number of individuals, *A* = number of alleles, *A*_r_ = allele richness, *H*_e_ = expected heterozygosity, *H*_o_ = observed heterozygosity, IR = internal relatedness, sMLH = standardized multilocus heterozygosity, HL = homozygosity weighted by locus, and TrioML = inbreeding index. *A*, *A*_r_, *H*_e_ and *H*_o_ were estimated by locus, whereas sMLH, HL, IR and TrioML are individual based estimates.

### Heterozygosity and inbreeding

**Figure 1:**
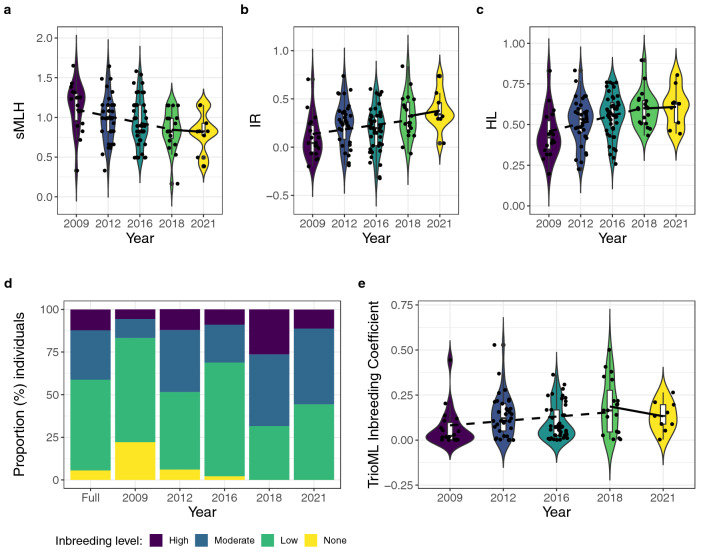
Violin plots showing temporal changes in heterozygosity and inbreeding in the captive peninsular pronghorn. Panels (**a**–**c**) show changes in three frequency-weighted measures of individual heterozygosity: standardized multilocus heterozygosity (sMLH), internal relatedness (IR) and homozygosity weighted by locus (HL) respectively. Panel (**d**) shows bar charts depicting the proportion (%) of individuals falling within different inbreeding classes, from none (*f* = 0), through low (*f* = < 0.125) and moderate (0.125 < *f* < 0.25) to high (*f* > 0.25), as estimated using TrioML. Panel (**e**) shows violin plots of changes in the inbreeding coefficient, estimated using TrioML. In panels (**a**–**c**) and (**e**), the boxplots span the first to third quartiles, with horizontal lines inside the boxes representing the medians. The raw data are plotted as black points and the lines connecting the boxplots correspond to regression lines smoothed and fitted with the “glm” function separately for the years 2009–2018 (dashed lines) and 2018–2021 (solid lines).

Three frequency-weighted microsatellite-based measures of individual heterozygosity—standardized multilocus heterozygosity (sMLH), internal relatedness (IR) and homozygosity weighted by locus (HL)—showed consistent trends of declining heterozygosity over time (all significant at *p* < 0.05, Table 2, Fig. 1a–c). Based on the TrioML inbreeding index, we found that the captive herd of the Peninsula pronghorn is moderately inbred, with *f* averaging 0.12 (95% SE = 0.01) and ranging from zero to 0.53 (Fig. 1d, Table 1). We also detected a significant increase in inbreeding over time (Table 2, Fig. 1e), which was mainly attributable to an increase in the proportion of moderately to highly inbred individuals (*f* > 0.125) from 16.6% in 2009 to 55.5% in 2021 (Fig. 1, Supplementary Table S6 online).

**Table 2:** Results of the generalized linear models (GLMs) of the effect of time on estimates of individual genetic diversity and the inbreeding index.

Diversity estimate	Time interval	Number of observations	Estimate (SE)	*p* value	Intercept (SE)
sMLH	2009–2021	124	− 0.08 (0.02)	**0.001**	1.16 (0.07)
	2009–2018	115	− 0.08 (0.03)	**0.004**	1.18 (0.08)
	2018–2021	28	− 0.02 (0.10)	0.79	0.95 (0.44)
IR	2009–2021	124	0.05 (0.02)	**0.003**	0.07 (0.05)
	2009–2018	115	0.05 (0.02)	**0.03**	0.09 (0.06)
	2018–2021	28	0.06 (0.09)	0.50	0.08 (0.37)
HL	2009–2021	124	0.04 (0.01)	**0.0001**	0.42 (0.03)
	2009–2018	115	0.05 (0.41)	**0.0003**	0.41 (0.04)
	2018–2021	28	0.009 (0.05)	0.83	0.56 (0.20)
TrioML	2009–2021	124	0.018 (0.009)	0.06	0.07 (0.02)
	2009–2018	115	0.024 (0.01)	**0.03**	0.06 (0.03)
	2018–2021	28	− 0.05 (0.05)	0.32	0.40 (0.23)

Significant *p* values are highlighted in bold.

### Temporal change in heterozygosity and inbreeding

To investigate whether the genetically informed breeding management attempt in 2018 could have helped to slow down the loss of heterozygosity, we implemented regressions of diversity estimates on time for the periods 2009–2021, 2009–2018 and 2018–2021 (Fig. 1, Table 2 and Supplementary Table S7 online). Almost all of the frequency-weighted measures of individual heterozygosity and inbreeding (sMLH, IR, HL and TrioML) showed significant temporal trends at *p* < 0.05 for the periods 2009–2018 and 2009–2021. The only exception was the TrioML inbreeding index over the period 2009–2021 (*p* = 0.06). Nevertheless, for the marker-based estimates (*A*_r_, *H*_o_ and *H*_e_) only *H*_*o*_ was significant and only for the period 2009–2018. None of the estimates were significant for the period 2018–2021 (Table 2 and Supplementary Table S7 online). Although we do not have a sufficiently long time series after the breeding management attempt to allow us to formally test for differences in the slopes, we did observe a tendency for the slopes to decrease after 2018, at least for sMLH and HL (Fig. 1a–c). Furthermore, the proportion of highly inbred offspring (*f* > 0.25) declined from 26.3% in 2018 to 11.1% in 2021 (Fig. 1d, Supplementary Table S6 online). Accordingly, the predicted values from the GLMs based on data from 2009 to 2018 projected a greater amount of genetic erosion than was actually observed (Table 3).

**Table 3:** Predicted values (from GLMs) of genetic diversity for the 2021 cohort assuming that no genetically informed breeding programme had taken place.

Diversity estimate	Empirical value for 2021 (lower and upper 95% CI)	Predicted value from a GLM for the period 2009 to 2018 (lower and upper 95% CI)
sMLH	0.82 (0.62–1.02)	0.76 (0.61–0.91)
IR	0.38 (0.23–0.53)	0.32 (0.20–0.44)
HL	0.61 (0.51–0.70)	0.66 (0.59–0.73)
TrioML	0.13 (0.07–0.19)	0.17 (0.11–0.24)

### Historical demography

**Figure 2:**
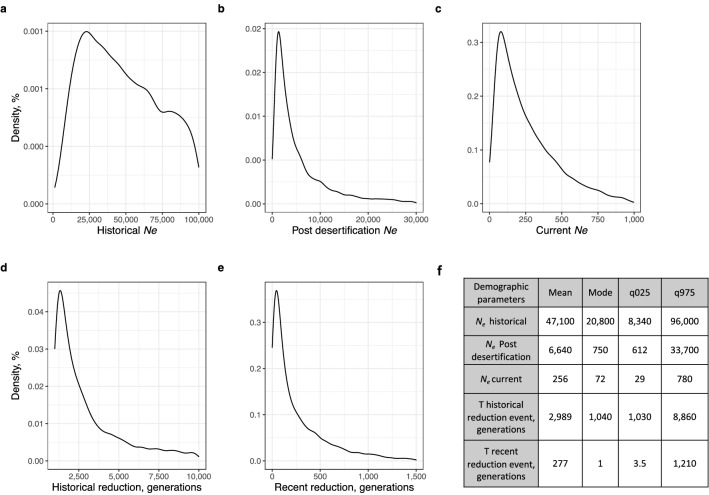
Posterior density curves and numerical estimates of demographic parameters for the best supported demographic scenario, which contains both a historical and a more recent reduction. (**a**) Historical effective population size; (**b**) effective population size before the recent demographic reduction; (**c**) current effective size of the captive peninsular pronghorn herd; (**d**) the number of generations since the historical demographic reduction; and (**e**) the number of generations since the recent demographic reduction. Panel (**f**) shows the mean, mode and 95% confidence intervals of each estimated demographic parameter.

We used approximate Bayesian computation (ABC) to evaluate four alternative historical demographic scenarios (Supplementary Fig. S2 online and Methods section for details). The best supported model (59%, CI = 58–60%) contained both a historical and a recent demographic reduction, while the second-best supported model (~ 33%) contained only a recent demographic reduction. The prior predictive error (i.e. the proportion of wrongly identified scenarios over 1000 test datasets drawn from a random sample of the chosen scenario) was high (logistic approach, 0.56) but the posterior error (i.e. the proportion of wrongly identified scenarios over 1000 test datasets drawn from the simulated datasets closest to the observed dataset) was lower at 0.39. Five demographic parameters were estimated for the best supported model (Fig. 2). Although posterior estimates for the contemporary and historical *N*_e_ were broad, we observed a large, over 200-fold decrease in the current *N*_e_ in comparison to the historical estimate (Fig. 2a–c). Assuming a pronghorn generation time of approximately two years^35^, we inferred that the first historical decline occurred approximately 6000 (95% CI 2060–17,720) years ago, whereas second decline appears to date back to around 554 (95% CI 7–2420) years ago (Fig. 2d,e).

## Discussion

Our time-series genetic dataset represents a new resource for the conservation management of the Peninsular pronghorn and has produced at least two significant discoveries. First, we uncovered a gradual erosion of the genetic diversity of the captive herd over time, although this trend appears to have partially abated in response to a genetically informed breeding management. Second, we could show that the genetic diversity of the Peninsular pronghorn has been shaped by a combination of historical and recent demographic changes driven by the ecological transformation of the Baja California peninsula and by anthropogenic pressures including hunting and habitat destruction. Below, we discuss the relevance of these findings to the Peninsula pronghorn conservation.

### Temporal changes in the genetic diversity of the captive herd

The Peninsular pronghorn experienced a severe decline over the last two centuries, from once being present across much of the Baja California peninsula to being functionally extinct in the wild^51,52,59^. This decline motivated the captive breeding program initiative^44,53^. In spite of early difficulties related to management, health problems and juvenile mortality^64^, by the end of 2021 and with approximately 700 individuals, the Peninsular Pronghorn Conservation Programme achieved this goal^53^. Until now, however, we lacked an understanding of how the last 13 years of captivity may have shaped the genetic composition of the captive herd.

Although we did not find any statistically significant temporal changes in several marker-based estimates of genetic diversity (*A*_r_, *H*_e_ and *H*_o_), a tendency for reduction was observed for *H*_e_ (9.8%, from 0.51 in 2009 to 0.46 in 2021) and *H*_o_ (16.3%, from 0.55 in 2009 to 0.46 in 2021). Furthermore, all three frequency-based estimates of individual heterozygosity (sMLH, IR and HL) revealed significant decreases in heterozygosity during the course of the study with, for example, sMLH falling by around 27% over the past 13 years. This pattern was mirrored by the TrioML based inbreeding coefficient, which showed a gradual reduction in the number of non-inbred and weakly inbred individuals and a concurrent increase in the number of moderately to highly inbred individuals over time. These findings are consistent with the theoretical expectation of zygosity being a function of the breeding system^65^. Thus, inbreeding directly reduces heterozygosity by increasing the proportion of homozygotes relative to random expectations, but only indirectly affects allelic richness. By contrast, genetic drift directly affects allelic diversity but only indirectly impacts heterozygosity^66,67^. Therefore, our results suggest that inbreeding is currently the predominant force shaping the genetic diversity of the Peninsular pronghorn.

The genetic management of captive populations has proven to be extremely effective in preventing the loss of genetic diversity and ameliorating the negative effects of inbreeding^68–70^. Accordingly, we found that the implementation of a genetically informed breeding management attempt that have begun in 2018 was associated with a slight reduction in the slope of the relationship between heterozygosity and time, as well as with a reduction in the proportion of highly inbred individuals. Furthermore, model-based predictions suggest that the 2021 offspring cohort is significantly more outbred than would be expected if no genetic management had been undertaken. Consequently, although it is still rather early to tell, our preliminary results suggest that this strategy might be beneficial in terms of mitigating inbreeding and the loss of heterozygosity.

### Limitations of our study

While our results provide grounds for cautious optimism, a number of caveats should be born in mind. First, in our study, comparisons among different years were not always based on the same age class, with only fawns being sampled in 2012, 2016 and 2021, and adults being sampled in 2009 and 2018. However, the difficulty of handling captive pronghorn meant that it was not possible to exhaustively sample both age classes across all years. We therefore focused on sampling adults at the beginning of the study and in the year of the breeding management attempt in order to provide reference populations against which subsequent generations of offspring could be compared. Furthermore, estimates of inbreeding increased between adults sampled in 2009 and 2018, while the offspring cohorts sampled in 2012, 2016 and 2021 also showed trends of decreasing heterozygosity and increasing inbreeding over time.

A second important caveat is that our sample size of individuals was modest (*n* = 144) in comparison to the total size of the captive herd (approximately 700 individuals). Incomplete sampling may be particularly important regarding the outcomes of management actions, as the effect of breeding recommendations will not be fully realised if animals with unknown relationships are allowed to mate as well as animals of known kinship. Consequently, the reduction in the loss of diversity that we observed after the implementation of the genetically informed breeding attempt may be conservative in the sense that more thorough sampling might have produced an even better outcome. Regardless, given that it is not possible to provide breeding recommendations for unsampled individuals, a strong case can be made for increasing sampling rates into the future. This would help to further optimise partner selection and thereby ensure the best possible retention of genetic diversity and reduction of inbreeding. Larger sample sizes of genotyped individuals would also be beneficial for the ongoing monitoring of genetic changes within the captive population.

Third, previous studies of the American pronghorn subspecies have documented inbreeding depression for multiple traits from birth mass through fawn survival to body condition^71^. However, fitness data have not yet been systematically collected for the Peninsular pronghorn, precluding an analysis of the potential negative effects of inbreeding in this subspecies. Consequently, future studies should aim to quantify the magnitude of inbreeding depression in the captive Peninsula pronghorn population, as well as to evaluate whether temporal changes in the amount of inbreeding are associated with changes in the mean fitness of the population. The simplest approach for this would be to test for associations between heterozygosity and fitness components such as fawn body mass and survival, although it would be preferable to estimate inbreeding more reliably using population genomic approaches such as reduced representation sequencing^72^.

Fourth, over longer timescales, a handful of microsatellites cannot tell us very much about the nature or magnitude of functional genetic variation, although neutral genetic diversity should in general provide a rough indication of the adaptive potential of a given species^73^. Nonetheless, we believe that the recent sequencing of the pronghorn genome^74^ will facilitate more detailed investigation of how unintended selection and drift may have impacted the genetic composition of the Peninsula pronghorn. In particular, dense single nucleotide polymorphisms mapped to the reference genome could be used to characterize selective sweeps as well as runs of homozygosity, identical by descent haplotypes that are informative both about inbreeding and population history^75^. Furthermore, computational approaches have been developed to infer the presence of putatively deleterious alleles from whole genome resequencing data^76,77^. Application of these approaches to pronghorn would shed light on the mutation load and its relationship to historical demography both within and across species.

Finally, it should be born in mind that developing a larger breeding programme that extends to the entire captive pronghorn herd would bring significant challenges. Currently, the Peninsular pronghorn population is kept in large enclosures, with the smallest of these housing the pregnant females, spanning a total of around 100 ha. Therefore, the size of the pens places limits on the scope of intensive management actions. The captive handling of pronghorns also carries an increased risk of serious injury to the animals, especially as this species is susceptible to capture myopathy^78–80^. Finally, captive breeding and genetic management require access to financial resources^81^. Consequently, pronghorn managers will need to weigh all of the pros and cons to design and implement a genetic management programme that is optimized for this species and which is feasible given financial and logistical constraints. For example, group-based management actions may be a good alternative to individual-based management actions. Specifically, breeding females could be distributed among management units and among smaller pens within each unit. Small numbers of breeding males could subsequently be moved between pens to maintain gene flow. A single breeding male could potentially breed for several seasons in different pens and management units before being replaced^35^.

### Historical demography

Characterizing the strength and timing of historical declines can provide insights into the causes of those declines and thereby help conservation practitioners to create conditions that promote population recovery^82^. Based on our demographic reconstruction of the Peninsular pronghorn, we inferred that the onset of the decline may have been linked to climatic changes at the end of the LGM and the ensuing desertification of the Baja California peninsula. This is not surprising given that the contraction of open woodlands and expansion of desert scrub after the last glaciation are believed to be responsible for multiple extinction events as well as shifts in the geographical distributions of many animal species on the Baja California peninsula^83–85^. Furthermore, droughts have been recognized as one of the most important factors affecting the recruitment, mortality and abundance of pronghorns in arid and semi-arid areas^86–88^. For example, a devastating drought in 2002 reduced the number of Sonoran pronghorns in the USA to just 21 animals, motivating a captive breeding initiative as well as the introduction of animals from Mexico^48,89^. Consequently, our findings are consistent with the argument that precipitation is one of the most important factors limiting the abundance and geographical distribution of pronghorn^62^.

Our demographic analysis also uncovered evidence for a more recent demographic decline dating back around five hundred years ago. This is supported by recent studies showing that Peninsular pronghorn numbers have decreased to fewer than 100–150 individuals over the past hundred years or so^44,51^. Our results therefore point towards a scenario involving two consecutive declines, the first mediated by climate related vegetational changes on the Baja California peninsula and the second driven by increasing anthropogenic pressures such as hunting, fencing and cattle ranching. Both climatic and anthropogenic stressors will likely continue to be significant threats to the Peninsular pronghorn over the coming decades^44,53,62^. In this regard, species abundance models could be a useful tool for identifying suitable areas for future reintroductions based on a combination of human threats and climatic projections. Moreover, genetic information could be used to optimally select individuals for release in such a way as to minimize inbreeding and maximise genetic diversity^7,90^.

To conclude, we investigated changes in heterozygosity and inbreeding over time in the captive Peninsular pronghorn herd and used demographic reconstruction to evaluate alternative hypotheses relating to the decline of this subspecies. We found that, although the captive population has become progressively more inbred over time, genetically informed management appears to have partially counteracted this trend. We could also show that the Peninsular pronghorn likely experienced a gradual, protracted decline with two consecutive phases linked respectively to environmental change and anthropogenic impacts. Although the Peninsular pronghorn still faces multiple threats, the success of the captive breeding programme at building a large and demographically stable population may hint at unexpected resilience, and is a clear testament to the success of ongoing protection measures.

## Methods

### Research permissions and ethical considerations

All samples were collected by the management team of the Peninsular Pronghorn Conservation Programme under the registration key DGVS‐UMA‐VL‐3755‐BC given to the management unit by the Mexican Secretariat of Environment and Natural Resources. All procedures were approved by the authorized personal of the Valle de los Cirios Flora and Fauna Protection Area and followed the guidelines of the American Society of Mammalogists (www.mammalsociety.org/uploads/committee_files/CurrentGuidelines.pdf, accessed 7 January 2022). This work did not require any approval from the ethical committee since no experiments on live animals were performed, aside from the routine tagging that was performed by trained personnel and according to the conservation programme internal schedule. All procedures were in compliance with the ARRIVE guidelines for how to report animal experiments^91^.

### Sample collection

Tissue samples were collected from 144 peninsular pronghorn individuals by trained personnel during 2009–2021 from the Vizcaino Biosphere Reserve and Valle de los Cirios Flora and Fauna Protection Area (Supplementary Table S1 online). Small pieces of ear tissue were taken during the tagging and from deceased animals whenever those were found by the management team. Tissue samples were preserved in 100% ethanol at room temperature until processing. Samples were mainly taken from young individuals (newborns to animals up to 6 months of age) and occasionally from adults in 2012, 2016 and 2021, while in 2009 adults were sampled as a reference group. We additionally sampled adults in 2018 as part the breeding management attempt described above. Those individuals formed part of the breeding herd, with unrelated individuals preferentially selected as mating partners. Consequently, our sampling scheme spans 13 consecutive years out of the 23 years of the captive breeding programme.

### Molecular techniques

DNA extractions were performed using DNeasy Blood and Tissue Kit (Qiagen Inc., Valencia CA, USA) following the manufacturer’s protocol. DNA concentration was determined using a NanoDrop2000 (Thermo Scientific™) and each extract was adjusted to a concentration of approximately 100 ng/µl. We amplified 16 microsatellite loci previously described for pronghorn^92–94^ using the M13 genotyping approach^95^. Additionally, 12 samples were independently re-genotyped at eight microsatellites in order to estimate our genotyping error rate. Polymerase chain reactions were carried out in an 11.5 µL volume containing 1 µL of the DNA template, 1 × buffer (Invitrogen, Carlsbad, California), 1 mM MgCl_2_, 0.2 mM deoxynucleoside triphosphates, 0.05% bovine serum albumin, 0.5 U of Taq DNA Polymerase (Invitrogen), and 0.5 µM of each primer. The polymerase chain reaction profile consisted of an initial denaturalization step at 95 °C for 5 min, followed by 35 cycles of 60 s each at 95 °C, annealing by ramping from 55 to 60 °C, followed by 60 s extension at 72 °C. Cycles were terminated with a final extension stage of 10 min at 72 °C. PCR products were resolved on an Applied Biosystems 3730XL capillary sequencer at the University of Arizona Gene Core Facility and alleles were scored using PeakScanner v1.0 (Applied Biosystems).

### Data analysis

Genotypes were binned to size classes using FlexiBin^96^. After that, we imported the binned microsatellite data into the R environment^97^ (R version 4.1.2) and converted it into a GENIND object using *adegenet*^98^. We quantified the amount of missing data per locus and per individual using the R package *poppr*^99^ and removed all individuals that failed to genotype at four or more loci. We also used package *PopGenReport*^100^ to estimate null allele frequencies. To test for linkage disequilibrium (LD) between pairs of loci, we used *poppr*. For this analysis, we used the standardized index of association (rbarD)^101^ and the number of permutations was specified using the ‘sample = 999’ argument. We further tested for Hardy–Weinberg equilibrium using the *pegas* package^102^. Finally, in order to determine the uniqueness of the genotypes we used “mlg” function as implemented in *poppr*. All of the above analyses were performed on the complete dataset and separately for each year. Whenever multiple tests we used, the resulting *p*-values were adjusted for the false discovery rate (FDR) using the R package *stats*^97^.

### Genetic diversity and summary statistics

The number of alleles (*A*), allelic richness (*A*_*r*_), expected heterozygosity (*H*_*e*_) and observed heterozygosity (*H*_*o*_) were calculated for the full dataset and separately for each year using the R packages *adegenet*^98^ and *hierfstat*^103^. Multilocus heterozygosity was quantified as standardized multilocus heterozygosity (sMLH), internal relatedness (IR) and homozygosity weighted by locus (HL)^104^ using R packages *Rhh*^105^ and *inbreedR*^105,106^. We used COANCESTRY v. 1.0^107^ to calculate individual inbreeding coefficient using the TrioML method^108^, using 10,000 reference individuals and bootstrapping on 10,000 samples. Following Marshall et al.^109^, we designated inbreeding coefficients (*f*) of zero as ‘none’, below 0.125 as ‘low’, 0.125 ≥ *f* < 0.25 as ‘moderate’, and *f* ≥ 0.25 as ‘high’. Wilcox tests were then used to test for significant differences in the diversity indices using the R package *stats*. We also used generalized linear models (GLMs) to quantify the strength of diversity decline over the years (2009–2021, 2009–2018 and 2018–2021) using the R package *lme4*^110^. Finally, using the “predict” function in the R package *stats* and the slope of the GLM spanning 2009–2018, we determined the modeled value for each diversity estimate assuming that no genetically based breeding management attempt had been implemented.

### Demographic reconstruction

To investigate the demographic history of the Peninsular pronghorn, we used approximate Bayesian computation (ABC) as implemented in DIYABC v. 2.0^63,111,112^. For modeling alternative demographic histories and reconstructing demographic trajectories, we used data from 58 samples (fawns and adults) from 2016, which was the year represented by the largest number of individuals.

ABC allows the evaluation of alternative demographic scenarios, expressed as a stepwise series of population size changes, and then uses summary statistics from the observed and simulated datasets to estimate parameter values and to assess the relative support for each scenario. We first developed four alternative demographic models intended to describe plausible patterns of effective population size (*N*_e_) change over time. The mutation rate was set to range between 1e^−2^ and 1e^−5^. Priors for the timing of events and the magnitude of changes of *N*_e_ (Supplementary Fig. S2 online and Supplementary Table S8 online) were based on prior knowledge of the factors likely shaping the demographic history of the species, including environmental change on the Baja California peninsula after the LGM, anthropogenically induced population reduction and the captive breeding programme^42,60,61^. The first scenario represented the null hypothesis of (a) constant *N*_e_ over time; the alternative scenarios invoked: (b) a recent reduction caused by overexploitation and habitat loss, (c) a historical reduction caused by the desertification of the Baja California peninsula, and (d) a combination of recent and historical reductions, expressed as a two-step model (Supplementary Fig. S2 online). After simulating one million datasets for each scenario, we used a polychotomous logistic regression procedure^113^ to estimate the posterior probability of each scenario based on the 1% of simulated data sets for each model that produced summary statistics closest to the observed values. The error rate was estimated using prior data space and the posterior distributions. The posterior error rate represents the proportion of wrongly identified scenarios over the 1000 test datasets^63^. Based on the best supported scenario, local linear regression was used to estimate the posterior distributions of the parameters. Specifically, a logit transformation of parameter values was performed and the 1% closest simulated datasets to the observed were used for regression and posterior parameter estimation^113^.

## Supplementary Information

Supplementary Information.

## Data Availability

Our microsatellite dataset is available from the corresponding author on request or from Zenodo repository, https://doi.org/10.5281/zenodo.6014746.
