# Supplementary material for: The genetic consequences of captive breeding, environmental change and human exploitation in the endangered peninsular pronghorn
Source: Sci Rep. 2022 Jul 4;12:11253. doi: 10.1038/s41598-022-14468-4 (PMC9253347; doi:10.1038/s41598-022-14468-4)
Supplement: Supplementary file 1 — Supplementary Information. [file 41598_2022_14468_MOESM1_ESM.pdf]

Scientific Reports

Supplementary Information

"The genetic consequences of captive breeding, environmental change and human exploitation in the endangered peninsular pronghorn"

Anastasia Klimova, Jesús Gutiérrez-Rivera Victor Sanchez-Sotomayor, Joseph Ivan Hoffman

Supplementary figures and tables

Table of contents:

Supplementary figures Page 2

Supplementary tables Page 4

Supplementary figures.

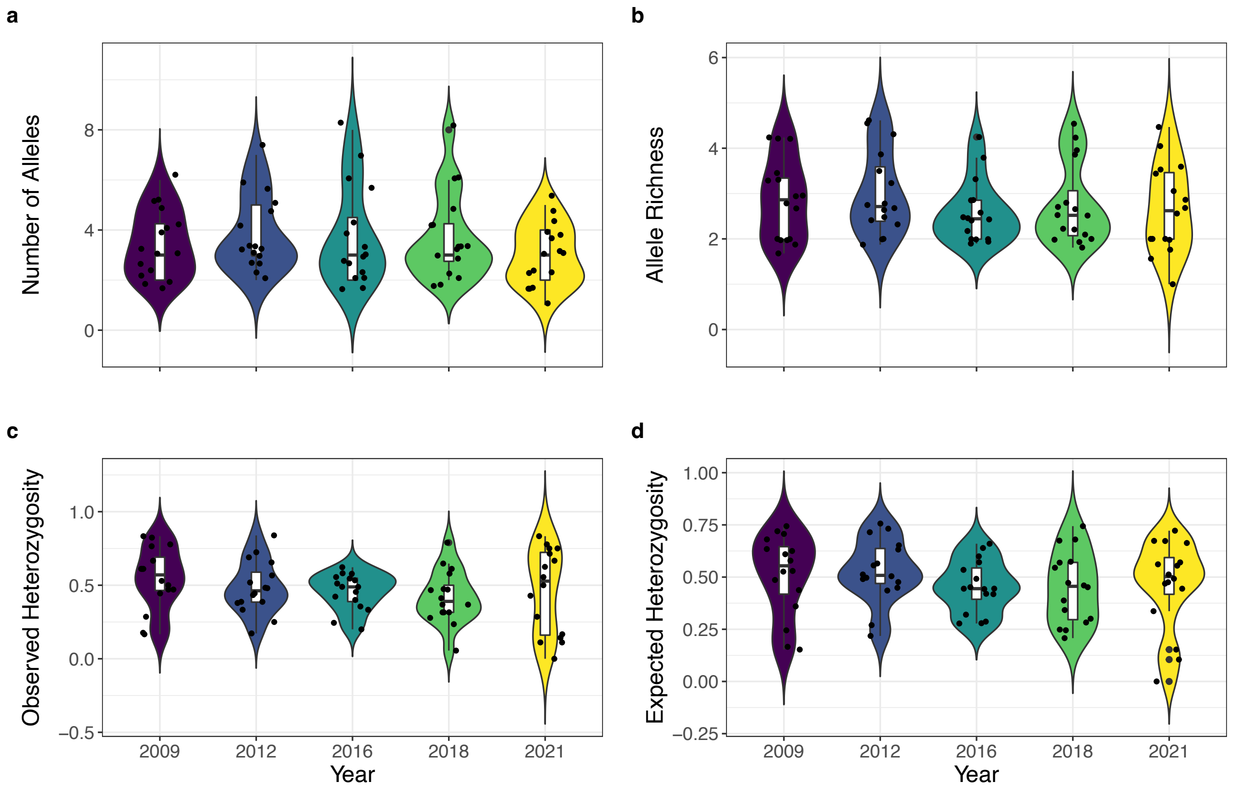

Supplementary Figure S1. Violin plots representing basic diversity estimates: (a) Number of Alleles; (b) Allelic Richness; (c) Observed and (d) Expected Heterozygosity for the captive peninsular pronghorn herd. Boxplots span the first to third quartiles, with horizontal lines inside the boxes representing the medians. The raw data are plotted as black points. No significant differences were found between any of the groups.

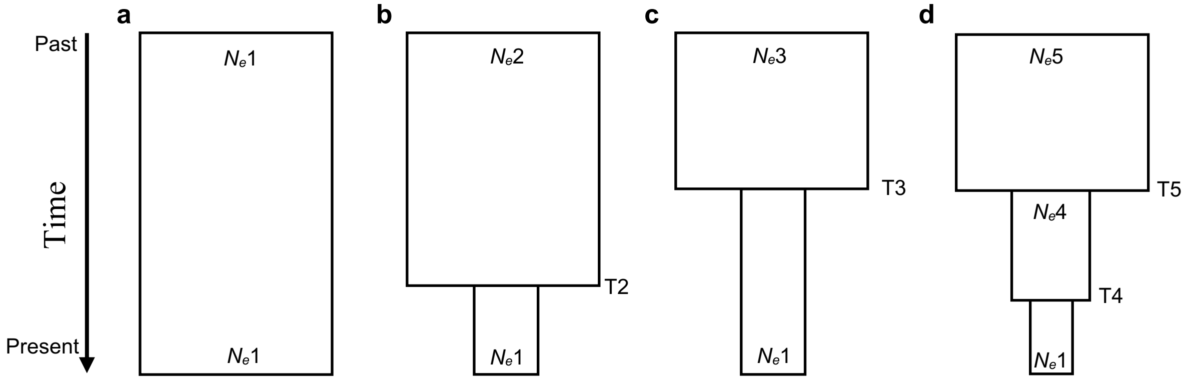

Supplementary Figure S2. Demographic scenarios tested using DIYABC. (a) Constant population size model: the effective population size was held constant at N_e1 from the past to the present. (b) Recent population reduction model: the effective population size was reduced from N_e2 to N_e1 at T2. (c) Historical population reduction model: the effective population size was reduced from N_e3 to N_e1 at T3. (d) Two-step population reduction model: the effective population size was reduced from N_e5 to N_e4 at T5 and then again from N_e4 to N_e1 at T4. In all scenarios, T represents the timescale measured in the number of generations.

Supplementary tables.

Supplementary Table S1. Details of the Peninsula pronghorn samples used in this study.

Year sampled	Number of sampled individuals	Age
2009	18	Adults (over 1 year old)
2012	37	Fawns (less than year old)
2016	45+13	Fawns (less than year old) + adults (over 1 year old)
2018	19	Young adults (over 1 year old)
2021	12	Fawns (less than year old)
Total	144	

Supplementary Table S2. Details of Hardy-Weinberg equilibrium (HWE) tests for the 16 microsatellite loci used in this study. Tests were performed on the complete dataset as well as separately for each year. Loci that remained significant after FDR correction are highlighted in bold.

Locus	Full dataset	2009	2012	2016	2018	2021
Aam13	**0.000**	1	0.06	0.16	**0.05**	0.32
Aam15	0.67	1	0.97	0.93	0.12	0.94
Aam6	**0.000**	**0.000**	0.08	0.20	1.00	0.94
Aam9	0.06	1	0.85	**0.00**	**0.00**	0.99
Aam11	0.29	1	0.33	0.86	0.58	0.40
Anam88	0.55	1	0.06	0.48	1.00	1.00
Aam2	**0.04**	0.52	0.06	0.08	0.85	1.00
Aam3	0.13	1	0.94	0.60	1.00	0.94
Aam4	0.47	1	0.33	0.93	0.85	1.00
Aam7	0.06	0.32	0.06	0.48	0.85	0.94
Aam5	1	1	0.18	0.45	1.00	1.00
Anam99	0.31	1	0.63	0.86	0.85	0.94
Anam24	**0.003**	1	0.14	0.53	0.58	1.00
Anam69	**0.000**	**0.000**	0.70	0.64	**0.05**	0.69
Anam50	0.91	1	0.06	0.60	0.58	0.94
Anam82	**0.000**	1	0.09	0.22	0.16	1.00

Supplementary Table S3. Null allele frequency estimates for 16 microsatellite loci used in this study. Tests were performed on the full dataset as well as separately for each year. Results for which 2.5th percentile did not include zero are highlighted in bold.

Locus	Observed frequency (2.5th–97.5th percentile)					
	Full dataset	2009	2012	2016	2018	2021
Aam13	**0.15 (0.06,0.23)**	0.06 (-0.09,0.26)	0.13 (-0.01,0.31)	0.03 (-0.02,0.1)	0.18 (-0.01,0.36)	0.33 (0,0.49)
Aam15	-0.01 (-0.06,0.06)	-0.11 (-0.2,0.02)	-0.03 (-0.13,0.07)	0.02 (-0.07,0.1)	-0.01 (-0.14,0.18)	0.04 (-0.14,0.3)
Aam6	**0.15 (0.08,0.24)**	0.18 (-0.03,0.45)	0.1 (-0.07,0.31)	0.07 (-0.01,0.16)	-0.05 (-0.11,-0.01)	-0.16 (-0.25,-0.06)
Aam9	0.03 (-0.02,0.07	-0.08 (-0.16,-0.01)	0.03 (-0.08,0.15)	0.02 (-0.03,0.08)	**0.23 (0.08,0.38)**	0 (-0.15,0.19)
Aam11	0.01 (-0.04,0.06)	-0.05 (-0.15,0.07)	-0.08 (-0.15,0)	-0.02 (-0.09,0.05)	0.07 (-0.06,0.21)	0.29 (-0.05,0.61)
Anam88	0.02 (-0.06,0.11)	-0.08 (-0.16,-0.02)	0.23 (0,0.49)	-0.06 (-0.1,-0.03)	-0.02 (-0.07,0)	NA
Aam2	0.05 (0,0.11)	-0.03 (-0.16,0.1)	**0.16 (0.02,0.33)**	0.04 (-0.04,0.12)	-0.06 (-0.15,0.02)	-0.06 (-0.19,0.1)
Aam3	0.01 (-0.05,0.08)	-0.07 (-0.2,0.1)	0.01 (-0.13,0.18)	-0.03 (-0.11,0.05)	-0.03 (-0.07,0)	-0.02 (-0.19,0.17)
Aam4	-0.01 (-0.07,0.05)	-0.03 (-0.07,-0.01)	0.06 (-0.08,0.23)	-0.03 (-0.08,0.03)	0.02 (-0.08,0.16)	-0.07 (-0.15,-0.02)
Aam7	0.03 (-0.04,0.1)	0.01 (-0.15,0.2)	0.11 (-0.07,0.33)	0.07 (-0.02,0.17)	0.05 (-0.08,0.22)	-0.15 (-0.25-0.07)
Aam5	-0.02 (-0.09,0.06)	-0.13 (-0.22 -0.06)	-0.13 (-0.25,0.01)	0.07 (-0.03,0.18)	-0.02 (-0.07,0)	-0.08 (-0.25,0.16)
Anam99	0.03 (-0.04,0.12)	-0.11 (-0.26,0.06)	0.04 (-0.1,0.35)	0.02 (-0.07,0.12)	-0.04 (-0.16,0.09)	0.09 (-0.15,0.38)
Anam24	-0.03 (-0.1,0.04)	-0.09 (-0.22,0.13)	0.05 (-0.12,0.27)	-0.04 (-0.08-0.02)	0 (-0.13,0.16)	-0.01 (-0.1,0)
Anam69	0.07 (0,0.13)	0.1 (-0.05,0.3)	-0.09 (-0.19,0.05)	0 (-0.08,0.09)	**0.19 (0.02,0.37)**	0.17 (-0.05,0.48)
Anam50	-0.03 (-0.08,0.02)	-0.03 (-0.17,0.17)	0.12 (-0.04,0.31)	-0.06 (-0.12,0)	-0.11 (-0.2,0.02)	-0.1 (-0.21,0.01)
Anam82	-0.06 (-0.11,0)	-0.04 (-0.09-0.01)	0.12 (-0.07,0.49)	-0.09 (-0.14,-0.03)	0.03 (-0.1,0.18)	-0.01 (-0.04,0)

Supplementary Table S4. Details of LD among 16 microsatellite loci used in this study. Tests were performed on the full dataset as well as separately for each year. Results for which the 2.5th percentile did not include zero were highlighted in bold.

	Full dataset	2009	2012	2016	2018	2021
rbarD	0.013	0.029	0.026	0.0001	-0.02	0.015
p-value	**0.02**	**0.01**	0.08	0.58	0.89	0.08

Supplementary Table S5. Details of the 16 microsatellite loci used in this study and their diversity characteristics in 124 Peninsular pronghorn individuals. *A* - number of alleles, H_e - expected heterozygosity, H_o - observed heterozygosity.

Locus	Reference	*A*	H_e	H_o
Aam13	Dunn et al. 2011	9	0.45	0.29
Aam15	Dunn et al. 2011	7	0.59	0.61
Aam6	Carling et al. 2003	3	0.51	0.36
Aam9	Dunn et al. 2011	8	0.71	0.64
Aam11	Dunn et al. 2011	10	0.66	0.62
Anam88	Mungui-Vega et al. 2013	3	0.34	0.31
Aam2	Carling et al. 2003	6	0.73	0.64
Aam3	Carling et al. 2003	6	0.49	0.51
Aam4	Carling et al. 2003	9	0.39	0.39
Aam7	Carling et al. 2003	4	0.58	0.49
Aam5	Carling et al. 2003	3	0.45	0.45
Anam99	Mungui-Vega et al. 2013	4	0.47	0.44
Anam24	Mungui-Vega et al. 2013	3	0.37	0.39
Anam69	Mungui-Vega et al. 2013	6	0.62	0.52
Anam50	Mungui-Vega et al. 2013	3	0.51	0.54
Anam82	Mungui-Vega et al. 2013	4	0.33	0.35
Overall		5.5	0.51	0.47

Supplementary Table S6. Proportion (%) of individuals falling within designated inbreeding classes, from no inbreeding ($f = 0$), to low ($f < 0.125$) through moderate ($0.125 < f < 0.25$) to high ($f > 0.25$), as estimated using TrioML.

Level of inbreeding	Sample set					
	Full dataset	2009	2012	2016	2018	2021
$f = 0$	5.6	22.2	6.1	2.2	0	0
$f < 0.125$	53.2	61.1	45.5	66.7	31.6	44.4
$0.125 < f < 0.25$	29.0	11.1	36.4	22.2	42.1	44.4
$f \geq 0.25$	12.1	5.6	12.1	8.9	26.3	11.1

Supplementary table S7. Results of the generalized linear models (GLMs) of the effect of years in captivity upon marker-based diversity estimates, based on different time intervals. Only significant *p*-values were highlighted in bold.

Diversity estimate	Time interval	Number of observations	Estimate (SE)	*p*-value	Intercept (SE)
Ar	2009-2021	80	-0.06 (0.06)	*0.36*	2.95 (0.23)
	2009-2018	64	-0.07 (0.09)	*0.46*	2.97 (0.26)
	2018-2021	32	-0.09 (0.33)	*0.78*	3.12 (1.49)
Ho	2009-2021	80	-0.02 (0.01)	*0.14*	1.54 (0.05)

	2009-2018<br>2018-2021	64<br>32	-0.04 (0.02)<br>0.04 (0.08)	***0.03***<br>*0.59*	1.58 (0.05)<br>1.24 (0.38)
He	2009-2021<br>2009-2018<br>2018-2021	80<br>64<br>32	-0.02 (0.01)<br>-0.03 (0.02)<br>0.01 (0.07)	*0.19*<br>*0.14*<br>*0.84*	1.53 (0.04)<br>1.55 (0.05)<br>1.39 (0.031)

Supplementary Table S8. Log uniform prior distributions used to estimate demographic parameters in DIYABC.

Parameter	Prior distribution	Condition
Size of the population at present (N_{e1})	[1 – 1,000]	
Size of the ancestral population before reduction (N_{e2}, N_{e4}), considering a recent anthropogenic reduction	[500 - 50,000]	$N_{e2} > N_{e1}$<br>$N_{e4} > N_{e1}$
Size of the ancestral population before reduction (N_{e3}), considering a historical, climate-related reduction	[500 - 1000,000]	$N_{e3} > N_{e1}$
Size of the second ancestral population, when two reduction events were considered (N_{e5})	[1,000-100,000]	$N_{e5}>N_{e4}$<br>$N_{e5}>N_{e1}$
Time point in generations of reduction event (T_2), considering a recent anthropogenic reduction	[1 - 2,000]	
Time point of reduction population event (T_3) considering a historical, climate-related reduction	[1,000 - 10,000]	
Time point of first (T_5) and second (T_4) reduction events	[1,000 - 10,000]<br>[1 - 2,000]	$T_4< T_5$
